# A Multi-Clade Test Supports the Intermediate Dispersal Model of Biogeography

**DOI:** 10.1371/journal.pone.0086780

**Published:** 2014-01-21

**Authors:** Ingi Agnarsson, Ren-Chung Cheng, Matjaž Kuntner

**Affiliations:** 1 Department of Biology, University of Vermont, Burlington, Vermont, United States of America; 2 Department of Entomology, National Museum of Natural History, Smithsonian Institution, Washington D.C., United States of America; 3 Institute of Biology, Scientific Research Centre, Slovenian Academy of Sciences and Arts, Ljubljana, Slovenia; 4 College of Life Sciences, Hubei University, Wuhan, Hubei, China; McGill University, Canada

## Abstract

**Background:**

Biogeography models typically focus on explaining patterns through island properties, such as size, complexity, age, and isolation. Such models explain variation in the richness of island biotas. Properties of the organisms themselves, such as their size, age, and dispersal abilities, in turn may explain which organisms come to occupy, and diversify across island archipelagos. Here, we restate and test the intermediate dispersal model (IDM) predicting peak diversity in clades of relatively intermediate dispersers.

**Methodology:**

We test the model through a review of terrestrial and freshwater organisms in the western Indian Ocean examining the correlation among species richness and three potential explanatory variables: dispersal ability quantified as the number of estimated dispersal events, average body size for animals, and clade age.

**Conclusions:**

Our study supports the IDM with dispersal ability being the best predictor of regional diversity among the explored variables. We find a weaker relationship between diversity and clade age, but not body size. Principally, we find that richness strongly and positively correlates with dispersal ability in poor to good dispersers while a prior study found a strong decrease in richness with increased dispersal ability among excellent dispersers. Both studies therefore support the intermediate dispersal model, especially when considered together. We note that many additional variables not here considered are at play. For example, some taxa may lose dispersal ability subsequent to island colonization and some poor dispersers have reached high diversity through within island radiations. Nevertheless, our findings highlight the fundamental importance of dispersal ability in explaining patterns of biodiversity generation across islands.

## Introduction

Models of island biogeography have long focused on the properties of islands, such as island size, age, characteristics, and isolation, and how these variables impact the probability of colonization, speciation, and extinction of organisms [1], [2]. These kind of models have proven incredibly powerful in explaining, for example, variation in patterns of species richness among islands [1], [3]. Rather less attention has been paid to how traits of organisms themselves, such as their size, age, and dispersal ability, may explain variation in those groups that tend to colonize and diversify on islands. Body size has been shown to correlate inversely with diversification rates in a variety of organisms [4] and may also impact probability of colonization, e.g. likelihood of being accidentally carried to an island by wind [5]. Clade age should, in general, positively correlate with richness [6], [7], though clade size over time will be determined by a complex interplay between speciation and extinction. Various other traits of organisms, such as reproductive mode, guild, trophic level, and many others may also explain patterns of diversification [8], [9], [10]. For island biogeography, dispersal ability seems an especially important parameter, as islands are surrounded by an oceanic matrix that can be a particularly effective barrier for many terrestrial organisms. Dispersal plays a key role in island colonization as well as determining gene flow among them, an interplay that must directly influence patterns of diversification [11], [12], [13], [14], [15], [16], [17].

Dispersal is one of the most fundamentally important biological attributes of organisms [5], and also one of principle mechanisms of inbreeding avoidance and of seeking suitable habitat patches [18]. Dispersal, however, incurs costs, both energetic and the risk of predation [19]. This trade off, combined with differences in the intrinsic properties of different organisms, leads to dramatic differences in dispersal propensity and ability of different taxa. Such differences have consequences at multiple scales, from individual fitness to population genetic structure and dynamics, to distribution and diversification of species and lineages [12], [20], [21]. At ecological time scales, meta-community diversity is predicted to peak at intermediate dispersal rates [11], [22]. However, dispersal also plays a crucial role in diversification patterns over evolutionary time where a key question is to determine the impact of dispersal distances and propensity on gene flow and speciation [5], [13], [15], [16], [17], [23], [24]. In sum dispersal abilities of organisms are expected to directly impact population structuring and the formation of new species. Dispersal ability not only promotes diversification through colonization of new landmasses that may be followed by evolutionary divergence, but at the same time constrains diversification via maintenance of gene flow among habitat patches/islands [15], [16], [17].

It is only recently that dispersal hypotheses again became focal parameters in island biogeography, as the accumulating molecular dating evidence tends to refute vicariance explanations for the distribution of many taxa [21], [25]. To date, nonetheless, focus has primarily been on the role of dispersal in determining distribution of lineages, in community assembly, and the evolutionary changes in dispersal propensity following island colonization [20], [23], [26], [27], [28], [29], [30]. Furthermore, the classical biogeography model of McArthur and Wilson [2] focused only on the role of dispersal in colonization, not in diversification through mediating or constraining gene flow. Very recently Claramunt et al. [15] formally restated the ‘intermediate dispersal model’ (IDM), building on ideas by earlier authors [16], [17], see also [14]. Claramunt et al. [15] focused on birds and thus mostly included good to excellent dispersers, but lacked poor and intermediate dispersers. This weakened their test. Here we provide a test of the IDM that includes primarily poor-intermediate-good dispersers of a variety of organisms, and thus complement the test of Claramunt et al.

### Hypothesis

**Figure 1:**
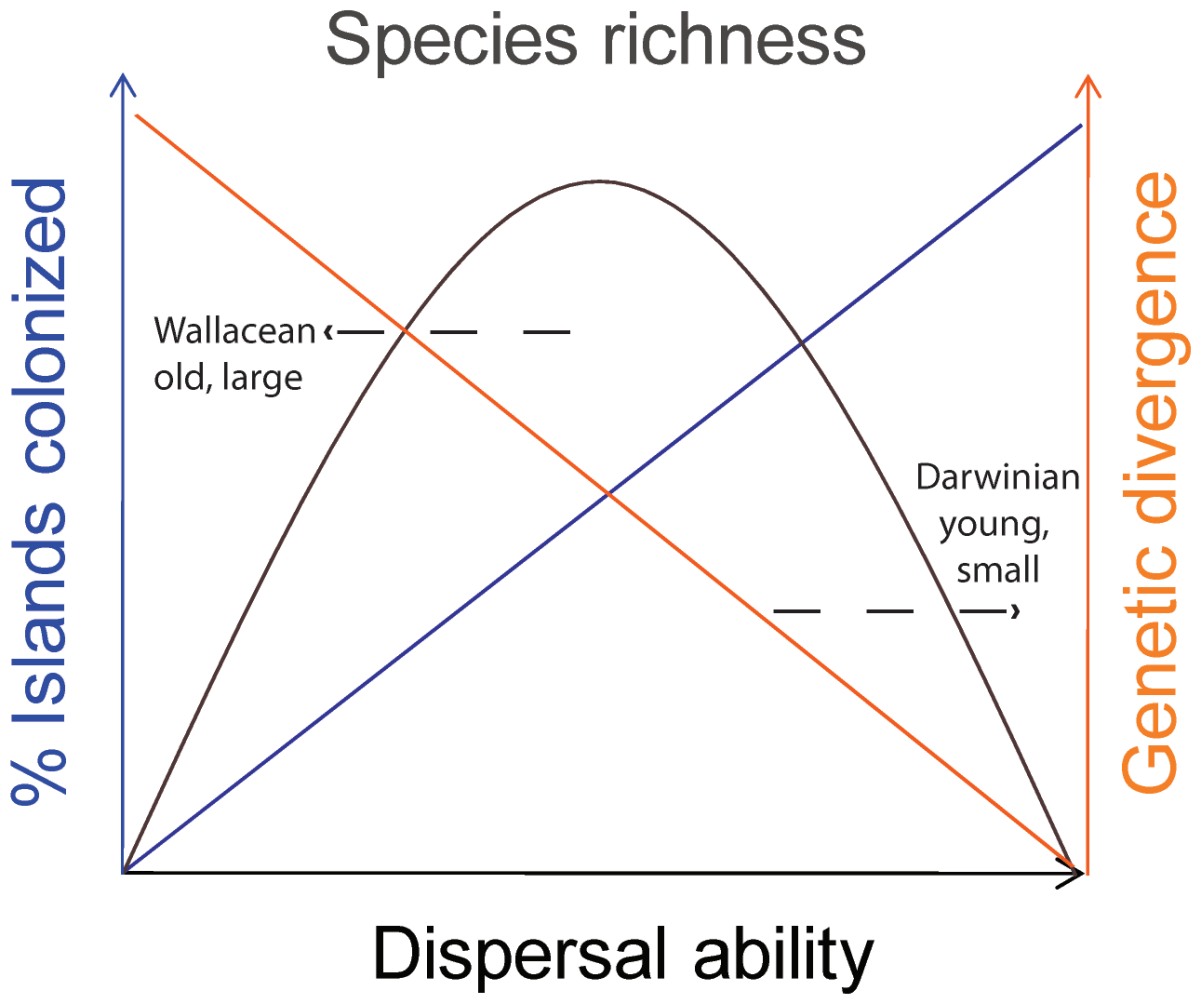
A graphical representation of the IDM. In co-distributed lineages of similar ages, dispersal ability (Fig. 1: x axis) positively, though not necessarily linearly as presented in this simple model, correlates with the number of isolated patches/landmasses a lineage will come to occupy (Fig. 1: y axis). On the other hand, dispersal ability will negatively, again not necessarily linearly, correlate with genetic divergence among populations (Fig. 1: z axis), as good dispersers maintain higher rates of gene flow [13]. The inevitable consequence of these two factors is the prediction that intermediate dispersers will be the most diverse: the poorest dispersers will not be able to colonize isolated landmasses, severely limiting opportunities for diversification, while excellent dispersers will repeatedly colonize many landmasses and maintain gene flow among them. Intermediate dispersers, however, have the opportunity to colonize new landmasses, but do so rarely enough so that colonization restricts gene flow among populations, leading to genetic divergence and eventually speciation [12]. A skew towards high diversity of relatively poorer dispersers is expected on large, old, Wallacean (fragment) islands, while small, young, Darwinian (de novo) islands will be home mostly to relatively good dispersers.

A simple conceptual model (Fig. 1, see also [15]) predicts that intermediate dispersers should be most diverse across habitat patches separated by barriers, such as island archipelagos [13]. Poor dispersers will colonize islands at very low rates and may be mostly absent on island archipelagos. However, gene flow is expected to be more rapidly disrupted in poor dispersers where even narrow barriers can be effective isolators. Hence, processes of allopatric speciation should operate earlier in poor dispersers, and they also have the potential to diversify within the few islands they come to occupy. On the other hand, excellent dispersers will be widespread, but are expected to maintain higher levels of gene flow, thus countering speciation, even among isolated and remote populations [15], [16], [17]. In intermediate dispersers, dispersal may be frequent enough to allow inter-island migration, but sufficiently limited to restrict gene flow and allow diversification [12]. Clearly then, dispersal is one of key parameters in the closely related disciplines of comparative phylogeography and island biogeography. Of course, other traits of organisms are also expected to impact their patterns of diversification, fundamental properties such as body size, generation times, and lineage age [4], [6], [7]. Comparing how well some of these different parameters explain species richness, as we do in a simple test below, can inform on their relative importance. IDM (Fig. 1) pointedly ignores all parameters other than dispersal ability, and thus to the extent that it is successful at explaining patterns of diversification, we believe it describes general trends that apply to organisms *despite* differences among them in a myriad of other traits.

Meta-analyses may provide a powerful way of testing the predictions from this model. A prior meta-analysis primarily tested the right side of the IDM curve (richness decreasing with dispersal ability) by focusing on good dispersers [15]. To complement that test we offer an analysis that primarily tests the left side of the curve (richness increasing with dispersal ability). Combining these tests, and including a variety of organisms should allow a general corroboration of the IDM.

## Methods

We reviewed recent literature on the biogeography of terrestrial and freshwater plant and animal taxa inhabiting the islands of the Western Indian Ocean [14]. We additionally collected data on lineage diversity (as regional species richness), estimated the minimal number of dispersal events and used it to quantify organisms’ dispersal ability, and also collected data on animal body size, and on clade ages, when available.

For the meta-analysis, we searched the Web of Science using the following literature search criteria: (Biogeography AND Madagascar OR Comoros OR Reunion OR Mauritius OR Rodrigues OR Socotra OR Seychelles OR Zanzibar) AND Year Published = (2006–2011); the hits were then refined by Web of Science categories: (Zoology OR Ecology OR Plant sciences OR Evolutionary biology) and by document type (Article OR Review), then further filtered by hand to retain only relevant works. The resulting Table (Appendix S1) lists those works that treat non-marine western Indian Ocean taxa explicitly and contain information on the phylogenetic relationships of those taxa. For these taxa, we extracted a biogeographically useful summary of the pattern of colonization and diversification in the archipelago. We obtained the number of species per group across the western Indian Ocean archipelago either directly from the biogeographical study or estimated it using additional resources, including both literature and online tools (e.g IUCN red list) (for additional detail see [14]). We also obtained the estimated number of dispersal events directly from papers when provided, but otherwise estimated it based on the phylogenetic results presented and the taxon distribution, where, in general, we assume as a minimum number of dispersal events equalling the number of islands occupied by a taxon. Additional events may be evidenced by the phylogeny, or fewer events in the case of lineages with vicariant histories. Because it was not possible to gage even an approximate number of dispersal events for the very strongest dispersers, we also categorized the data to be able to include these excellent dispersers in some of the analyses. We made arbitrary, but practical, categories ranging from 0 = extremely poor disperser for taxa where no dispersal events are inferred in the region, to 1 = poor for taxa with a single dispersal event, 2 = intermediate for taxa with 2–4 dispersal events, 3 = good for taxa with 5–30 dispersal events and 4 = excellent disperser for taxa showing regular inter-island dispersal. We also collected data on body size and clade ages, when available. Additionally, we include selected studies taken from older literature, and from the reviews of Yoder and Nowak [25] and Masters, de Wit, and Asher [31].

We used multiple regression in the statistical package R to explore which independent variables (dispersal ability, clade age, average body size for animals) affect the dependent one (diversity). To fit the assumptions of the method, we log transformed diversity and examined the normality of residuals. To quantify the influence of the independent variables for the model, we calculated the effect size by Cohen's ƒ^2^.

## Results and Discussion

The first analysis looking at the effect of body size was only done with animals and thus used a subset of all data, but since body size was found not to significantly affect diversity (P = 0.59), we used the full dataset for subsequent analyses focusing on dispersal ability and clade age.

**Figure 2:**
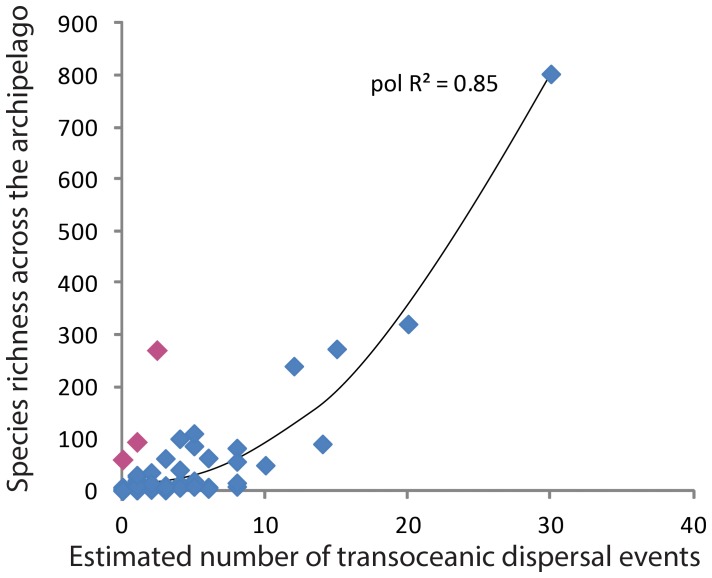
Meta-analysis as a preliminary test of the IDM: the richness of various clades of recently studied terrestrial and freshwater plants and animals in the western Indian Ocean, based on a review of nearly 100 recent studies [14]. Regression results show polynomial fit (R^2^ = 0.85, P<0.0001). For those taxa where dispersal event estimates are possible, diversity is largely explained by the number of estimated transoceanic dispersal events per lineage. Estimates of dispersal events are not available for excellent dispersers in the region. Nevertheless, clearly the best dispersers are both widespread and species poor (Fig. 3), implying that the relationship seen here breaks down at extreme dispersal abilities. Red dots show exceptionally diverse poor dispersers that all represent radiations on Madagascar either vicariant, or as a result of a single dispersal event. This highlights the extreme rarity of such events and thus how poor dispersal ability can severely restrict diversification among islands, but may promote it within the few islands–likely large and old–they by chance do colonize.

Our analysis found a correlation between diversity (species richness) and dispersal ability (P<0.001), and clade ages (P<0.001). However, dispersal ability affected diversity more than clade ages (ƒ^2^_dispersal ability_ = 0.67, ƒ^2^_clade age_ = 0.38). The use of multiple regression was justified with normally distributed residuals (W = 0.9765, P = 0.3091). In a simple regression analysis the number of estimated dispersal events explained 85% of the variation in diversity with the polynomial (R^2^ = 0.85, P<0.0001) being significantly better fit to the data than a linear regression (R^2^ = 0.69, P<0.0001, see Appendix S2, Fig. 2). These results corroborate the importance of dispersal ability in patterns of diversification among islands.

**Figure 3:**
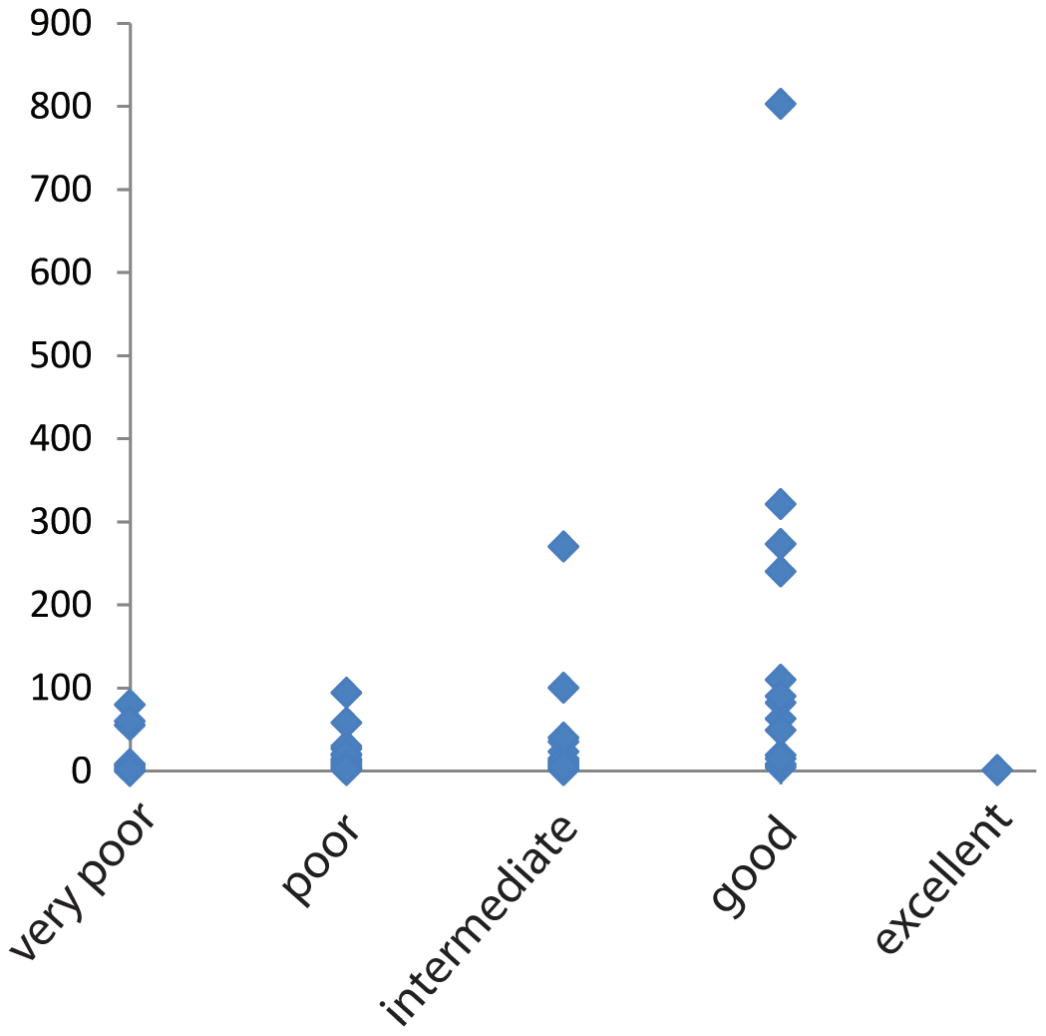
Species richness (on y) as a function of estimated dispersal ability. Rough categorical estimates of dispersal abilities were made from Appendix S1 based on frequency of transoceanic dispersal events ranging from very poor (zero estimated transoceanic dispersal events) to excellent (regular transoceanic dispersal). Clades that contain intermediate to good dispersers, that have undergone several transoceanic dispersal events, but do probably not maintain gene flow among islands, tend to be most species rich. Excellent dispersers are species poor, while poor dispersers range from species poor to moderately species rich, with the latter reflecting mostly within-island diversification in Madagascar. However, the exact number of such events or, for example, the minimum number of transoceanic dispersal events necessary to maintain gene flow and prevent speciation, is unknown but might be addressed through detailed genetic studies and/or simulations.

In the data covered by our review, most of the data come from poor to intermediate dispersers, with data mostly lacking from excellent dispersers. This limits our power to test the IDM. However, since the dataset analysed by Claramunt et al. [15] included good dispersers, both studies combined exhibit a good fit to the model. In our meta-analysis, the least diverse clades in the western Indian Ocean were found to be the very poor dispersers, which are largely or entirely absent on most islands, and the extremely good transoceanic dispersers, which are widespread, but species poor, as also found by Claramunt et al. [15], [25], [32]. As predicted, species richness per clade increases with dispersal ability (Fig. 2), however, at a certain point dispersal is frequent enough to maintain gene flow and prevent speciation, such that the best dispersers are species poor and the relationship between dispersal ability and species richness is that predicted by the IDM; bell-shaped (Fig. 3). Also seen is the predicted bias that within the large and old island of Madagascar, several lineages of relatively poor dispersers have colonized and diversified, sometimes extensively, within this island. Other groups are of Gondwanan origin in Madagascar and may be diverse without having ever dispersed over ocean. A difficulty of testing the model with empirical data is the problem of estimating the number of transoceanic events in excellent dispersers, such as the scooty tern, other oceanic birds, and certain dragonflies. Claramunt et al. [15] used hand-wing index to estimate dispersal ability of birds, but a single metric for organisms including insects, mammals, plants, fish, and birds, is more difficult to conceive. Our proxy of counting minimal dispersal events is coarse, but at least generalizable across all organisms. However, the more organisms disperse and interbreed among islands, the less trace of their dispersal history signature is left in their genes. Current molecular advances, such as next generation sequencing, however, will allow much more extensive genetic datasets for excellent dispersers, and thus offer an opportunity for future testing of the model.

### Confounding Factors

While our study, and that of Claramont et al. [15] corroborate the IDM, we nevertheless identify several potentially confounding factors. First, as stated above, relevant genetic data for excellent dispersers are difficult to obtain, and are currently largely unavailable. However, there is hardly any question that excellent dispersers such as *Pantala* dragonflies and *Onychoprion* terns maintain constant gene flow, and low diversity, among many islands. In fact, *Pantala* populations cross the western Indian Ocean annually [33]. Thus these are clearly two data points that would land within the lower right hypothetical area of Fig. 2. And, as Claramont et al. [15] used an index of dispersal independent of genetic data, and recovered the predicted patterns for excellent dispersers, the shortcomings of our data are less severe.

Second, geographical history, age, and size of islands will impact model predictions. Lineages that may have been present before the continental break-up (e.g. chameleons on Madagascar; [34]), or that may have arrived more recently through rare, stochastic dispersal events (e.g. radiation of Carnivora and Rodentia on Madagascar; e.g. [35]), may be diverse even though correctly characterized as poor dispersers. The diversification of poor dispersers within large and habitat rich islands might in some cases lead to higher diversity of dispersers characterized as poor than those characterized as ‘intermediate’. In fact, our choice of the western Indian Ocean including Madagascar may be one particularly likely to deviate from the model predictions as Madagascar could be characterized as a micro-continent, and is clearly an outlier among the remaining islands in the region. Such skews are evident in the empirical data (Fig. 2). On the other hand, we expect that Darwinian (de novo) islands, which have more recently emerged from the ocean (Fig. 1), will be home to mostly or entirely relatively good dispersers. Especially isolated Darwinian islands, e.g. Hawaii, will be colonized mostly by lineages with ancestors of excellent dispersing abilities [36]. If sufficient time is available, diversification may follow in part as a consequence of changes in dispersal ability subsequent to island colonization [36], [37].

Third, as a simplifying assumption, the current conceptual model ignores the issues of scale: A taxon may be relatively poor disperser at one scale, such as transoceanic dispersal between islands, and intermediate to good at another scale, such as within island dispersal. For example, in the context of western Indian Ocean at scales of hundreds of kilometres (Fig. 2), *Nephila* spiders are excellent dispersers and appear to maintain some gene flow among all the islands [38]. However, *Nephila* does not necessarily maintain gene flow among distant landmasses in the larger global context at the scale of thousands of kilometres. Many other organisms, however, are excellent dispersers even at global scales, for example many oceanic birds, cetaceans, and sea turtles. Accordingly, these taxa maintain gene flow globally and have speciated only modestly. A related issue are the intrinsic properties of organisms that impact their likelihood of within species diversification. Smaller animals, e.g. are more likely to speciate within small islands than are large animals.

Finally, another simplifying assumption is that we simply assumed the extinction rates to be relatively constant among different dispersers. How these confounding factors affect the predictions of the model remains to be established. It is remarkable, however, that the model has high explanatory power despite these confounding factors, an observation that suggests further and stronger testing of it will be worthwhile.

## Conclusions

To date the main focus on dispersal in biogeography has been its role in colonization and taxon distributions. However, the IDM predicts the fundamental importance of dispersal ability in shaping diversity patterns across archipelagos, or other habitat patches. The model receives support from an empirical dataset comparing a range of terrestrial and freshwater taxa throughout the western Indian Ocean that are mostly poor-intermediate/good dispersers, complementing a prior study focusing mostly on excellent dispersers [15]. The simple IDM pointedly ignores a number of variables and potential confounding factors, and does not claim to account for any diversification patterns other than those directly related to dispersal abilities. However, simplicity facilitates use, and critique, of models, and this basic model offers insight into the formation of biodiversity and the fundamental role that life history traits of organisms themselves, such as dispersal ability, can play in these evolutionary processes.

## Supporting Information

Appendix S1 **Estimated local diversity and number of dispersal events in the western Indian Ocean, in a little over 100 clades distributed, based on literature review 2005–2011.**
(DOC)

Appendix S2 **Results of regression analyses in R.**
(DOCX)
