# Supplementary material for: A Multi-Clade Test Supports the Intermediate Dispersal Model of Biogeography
Source: PLoS One. 2014 Jan 21;9(1):e86780. doi: 10.1371/journal.pone.0086780 (PMC3897756; doi:10.1371/journal.pone.0086780)
Supplement: Appendix S1 — Estimated local diversity and number of dispersal events in the western Indian Ocean, in a little over 100 clades distributed, based on literature review 2005–2011. (DOC) [file pone.0086780.s001.doc]

| **Kingdom** | **Taxon** | **Dispersal events** | **W Indian Ocean Diversity** | **Reference** |
| --- | --- | --- | --- | --- |
| animals | *Hemicordulia* dragonflies | 2 | 5 | Dijkstra (2007) |
| animals | Scops owls (*Otus*) | 3 | 7 | Fuchs et al. (2008) |
| animals | Parrots | 1 | 9 | Hume (2007) |
| animals | Parrots | 4 | 7 | Schweizer et al. (2010) |
| animals | Hawksmoth genus *Hyles* | 1 | 2 | Hundsdoerfer et al. (2009) |
| animals | Oscine passerine birds | 1 | n/a | Jonsson and Fjeldsa (2006) |
| animals | Golden orb spider (*Nephila inaurata*) | numerous | 1 | Kuntner and Agnarsson (2011) |
| animals | *Colotis* butterflies | 6 | 7 | Nazari et al. (2011) |
| animals | *Pteropus* fruit bats | 8 | 8 | O'Brien et al. (2009) |
| animals | *Drongos* (Dicruridae) | 6 | 4 | Pasquet et al. (2007) |
| animals | Carpenter bees (Ceratinini) | 2 | ? | Rehan et al. (2010) |
| animals | Allodapine bees | 2 | 35 | Schwarz et al. (2006) |
| animals | magpie robins (*Copsychus*) | 1 | 2 | Sheldon et al. (2009) |
| animals | Canthonini and Dichotomiini (Scarabaeinae) | n/a | n/a | Sole and Scholtz (2010) |
| animals | Scarabaeini beetles | 1 | 3 | Sole et al. (2011) |
| animals | White-eye songbirds (*Zosterops*) | 8 | 15 | Warren et al. (2006) |
| animals | Parrots | 1 | n/a | Wright et al. (2008) |
| animals | Starlings and mynas (*Sturnus,* *Acridotheres*) | 1 | 6 | Zuccon et al. (2008) |
| animals | *Rattus rattus* | n/a | 1 | Tollenaere et al. (2010) |
| animals | *Bactrocera* cucurbitae (Diptera: Tephritidae) | 0 | 1 | Virgilio et al. (2010) |
| animals | *Anelosimus* spiders | 4 | 12 | Agnarsson et al. (2010) |
| animals | *Mormopterus* bats | ? | 2 | Goodman et al. (2008) |
| animals | *Rousettus* bats | 2 | 2 | Goodman et al. (2010) |
| animals | *Chaerephon* bats | 1 | 2 | Goodman et al. (2010) |
| animals | Hermit spiders (*Nephilengys*) | 3 | 4 | Kuntner and Agnarsson (2011) |
| animals | *Drosophila mauritiana* | 1 | 1 | Legrand et al. (2011) |
| animals | *Triaenops* bats | 2 | 4 | Russell et al. (2008) |
| animals | *Scotophilus* bats | 2 | 4 | Trujillo et al. (2009) |
| animals | *Miniopterus* bats | below | below | Weyeneth et al. (2008) |
| animals | *Miniopterus* bats | 2 | 2 | Weyeneth et al. (2011) |
| animals | *Zaprionus* (Diptera) | 2 | 7 | Yassin et al. (2008) |
| animals | Geckos, skinks | 2 | 3 | Arnold and Bour (2008) |
| animals | *Leiolopisma* skinks | 2 | 3 | Austin and Arnold (2006) |
| animals | Slit-eared skinks | 2 | 3 | Austin et al. (2009) |
| animals | Haemadipisdae leeches | 3 | 8 | Borda and Siddall (2010) |
| animals | Ranid frogs | 3 | 270 | Bossuyt et al. (2006), Vieites et al. (2009) |
| animals | Freshwater crabs | below | below | Cumberlidge (2008) |
| animals | *Seychellum alluaudi* freshwater crab | below | below | Daniels (2011) |
| animals | Freshwater crabs | 3 | 15 | Daniels et al. (2006) |
| animals | *Homopholis* and *Blaesodactylus* gekkos | 2 | 4 | Greenbaum et al. (2007) |
| animals | Day geckos (*Phelsuma*) | 10 | 49 | Harmon et al. (2008) |
| animals | Day geckos (*Phelsuma*) | above | above | Raxworthy et al. (2007) |
| animals | *Pristionchus pacificus* nematode | ? | 1 | Herrmann et al. (2010) |
| animals | Knifefishes (Notopteridae) | 0 | 0 | Inoue et al. (2009) |
| animals | Gecarcinucoidea freshwater crabs | 5 | 19 | Klaus et al. (2006) |
| animals | River snails Pachychilidae | 1 | 5 | Kohler and Glaubrecht (2010) |
| animals | *Oplurin* iguanas | 1 | 7 | Munchenberg et al. (2008) |
| animals | *Stenophis* and *Lycodryas* snakes | 8 | 82 | Nagy et al. (2010) |
| animals | podocnemid turtles | 0 | 1 | Noonan and Chippindale (2006) |
| animals | iguanid lizards | 0 | 7 | Noonan and Chippindale (2006) |
| animals | Boid snakes | 0 | 3 | Noonan and Chippindale (2006) |
| animals | Agamidae | 0 | 0 | Okajima and Kumazawa (2010) |
| animals | Chamaeleonidae | 0 | 80 | Okajima and Kumazawa (2010) |
| animals | Plated lizards (Gerrhosauridae) | 1 | 19 | Raselimanana et al. (2009) |
| animals | Cetartiodactyla | 1 | 3 | Yoder and Nowak (2006) |
| animals | Eulipotyphla | 1 | 1 | Yoder and Nowak (2006) |
| animals | Rodents | 1 | 27 | Yoder and Nowak (2006) |
| animals | Carnivora | 1 | 9 | Yoder and Nowak (2006) |
| animals | Afrotheria | 1 | 30 | Yoder and Nowak (2006) |
| animals | Perissodactyla | 0 | 0 | IUCN |
| animals | Pholidota | 0 | 0 | IUCN |
| animals | Onychoprion | frequent | 1 | IUCN |
| animals | Pantala | annual | 1 | Anderson (2009) |
| animals | Primates | below | below | Heads (2010) |
| animals | Primates | 1 | 94 | Yoder and Nowak (2006) |
| animals | Coastal lizards (Cryptoblepharus) | 5 | Unresolved | Rocha et al. (2006) |
| animals | Mabuya lizards (Scincidae) | 4 | 14 | Rocha et al. (2010) |
| animals | Streptaxid land snails | 12 | 240 | Rowson et al. (2011) |
| animals | Crayfishes (Parastacidae) | 0 | 6 | Toon et al. (2010) |
| animals | *Archaius* (Calumma) chameleons | 1 | 1 | Townsend et al. (2011) |
| animals | Polystomatid flatworms (amphibian parasites) | 2 | 13 | Verneau et al. (2009) |
| animals | Blindsnakes (Typhlopidae) | 1 | 14 | Vidal et al. (2010) |
| animals | Giant pill-millipedes (Sphaerotheriida) | 0 |  | Wesene and VandenSpiegel (2009) |
| animals | Giant pill-millipedes (Arthrosphaeridae) | 0 | 60 | Wesener et al. (2010) |
| animals | Caecilian amphibians | 0 | 6 | Zhang and Wake (2009) |
| animals | Hemidactylus geckos | 2 | 11 | Carranza and Arnold (2006) |
| animals | *Chamaeleo monachus* | 0 | 1 | Macey et al. (2008) |
| plants | *Coffea* (Rubiaceae) | below | below | Anthony et al. (2010) |
| plants | Coffea (Rubiaceae) | below | below | Maurin et al. (2007) |
| plants | Coffee family (Rubiaceae) | 30 | 803 | Wikstrom et al. (2010) |
| plants | Chrysophylloideae (Sapotaceae) | 4 | 40 | Bartish et al. (2011) |
| plants | *Lilaeopsis* (Apiaceae subfamily Apioideae) | 0 | 1 | Bone et al. (2011) |
| plants | *Vanilla* | 3 | 10 | Bouetard et al. (2010) |
| plants | Sapindaceae (*Molinaea*, *Neotina*, *Tina,Tinopsis*) | 4 | 100 | Buerki et al. (2011) |
| plants | Palm tribe Chamaedoreeae | 3 | 5 | Cuenca et al. (2008) |
| plants | Scaly tree ferns | 8 | 56 | Janssen et al. (2008) |
| plants | Hibisceae (Malvaceae) | 5 | 86 | Koopman and Baum (2008) |
| plants | Fern genus *Platycerium* | 4 | 6 | Kreier and Schneider (2006) |
| plants | Euphorbs | 1 | 11 | Kulju et al. (2007) |
| plants | Dombeyoideae (Malvaceae) | 15 | 273 | Le Pechon et al. (2009) |
| plants | Dombeyoideae (Malvaceae) | above | above | Le Pechon et al. (2010) |
| plants | tropical Anagallis (Myrsinaceae) | 2 | 10 | Manns and Anderberg (2011) |
| plants | Hernandiaceae | 2 | 4 | Michalak et al. (2010) |
| plants | Angraecoid orchids | 20 | 321 | Micheneau et al. (2008) |
| plants | Calyptrochaeta moss | 3 | 1 | Pokorny (2011) |
| plants | Monimiaceae | 6 | 63 | Renner et al. (2010) |
| plants | *Lomariopsis* ferns | 3 | 7 | Rouhan et al. (2007) |
| plants | Cucurbitaceae | 14 | 90 | Schaefer et al. (2009) |
| plants | Ferns (Lindsaeaceae) | n/a | 31 | Lehtonen et al. (2010) |
| plants | *Warneckea* (Melastomataceae) | 1 | 20 | Stone and Andreasen (2010) |
| plants | *Aerva* (Amaranthaceae) | 5 | 8 | Thiv et al. (2006) |
| plants | Baobab *Adansonia digitata* | 1 | 1 | Tsy et al. (2009) |
| plants | *Diospyros* (Ebenaceae) | 2 | 14 | Venkatasamy et al. (2006) |
| plants | *Euphorbia* | 5 | 110 | Zimmermann et al. (2010) |
| plants | *Echidnopsis* (Apocynaceae) | 1 | 5 | Thiv and Meve (2007) |
| plants | *Thamnosma* (Rutaceae) | 1 | 2 | Thiv et al. (2011) |

1. Agnarsson I, Kuntner M, Coddington JA, Blackledge TA (2010) Shifting continents, not behaviours: independent colonization of solitary and subsocial Anelosimus spider lineages on Madagascar (Araneae, Theridiidae). Zoologica Scripta 39: 75-87.

2. Anthony F, Diniz LEC, Combes MC, Lashermes P (2010) Adaptive radiation in Coffea subgenus Coffea L. (Rubiaceae) in Africa and Madagascar. Plant Systematics and Evolution 285: 51-64.

3. Arnold EN (2009) Relationships, evolution and biogeography of Semaphore geckos, Pristurus (Squamata, Sphaerodactylidae) based on morphology. Zootaxa: 1-21.

4. Arnold EN, Bour R (2008) A new Nactus gecko (Gekkonidae) and a new Leiolopisma skink (Scincidae) from La Reunion, Indian Ocean, based on recent fossil remains and ancient DNA sequence. Zootaxa: 40-50.

5. Austin JJ, Arnold EN (2006) Using ancient and recent DNA to explore relationships of extinct and endangered Leiolopisma skinks (Reptilia : Scincidae) in the Mascarene islands. Molecular Phylogenetics and Evolution 39: 503-511.

6. Austin JJ, Arnold EN, Jones CG (2009) Interrelationships and history of the slit-eared skinks (Gongylomorphus, Scincidae) of the Mascarene islands, based on mitochondrial DNA and nuclear gene sequences. Zootaxa: 55-68.

7. Bartish IV, Antonelli A, Richardson JE, Swenson U (2011) Vicariance or long-distance dispersal: historical biogeography of the pantropical subfamily Chrysophylloideae (Sapotaceae). Journal of Biogeography 38: 177-190.

8. Bone TS, Downie SR, Affolter JM, Spalik K (2011) A Phylogenetic and Biogeographic Study of the Genus Lilaeopsis (Apiaceae tribe Oenantheae). Systematic Botany 36: 789-805.

9. Borda E, Siddall ME (2010) Insights into the evolutionary history of Indo-Pacific bloodfeeding terrestrial leeches (Hirudinida : Arhynchobdellida : Haemadipisdae). Invertebrate Systematics 24: 456-472.

10. Bossuyt F, Brown RM, Hillis DM, Cannatella DC, Milinkovitch MC (2006) Phylogeny and biogeography of a cosmopolitan frog radiation: Late cretaceous diversification resulted in continent-scale endemism in the family ranidae. Systematic Biology 55: 579-594.

11. Bouetard A, Lefeuvre P, Gigant R, Bory S, Pignal M, et al. (2010) Evidence of transoceanic dispersion of the genus Vanilla based on plastid DNA phylogenetic analysis. Molecular Phylogenetics and Evolution 55: 621-630.

12. Buerki S, Lowry PP, Andriambololonera S, Phillipson PB, Vary L, et al. (2011) How to kill two genera with one tree: clarifying generic circumscriptions in an endemic Malagasy clade of Sapindaceae. Botanical Journal of the Linnean Society 165: 223-234.

13. Cuenca A, Asmussen-Lange CB, Borchsenius F (2008) A dated phylogeny of the palm tribe Chamaedoreeae supports Eocene dispersal between Africa, North and South America. Molecular Phylogenetics and Evolution 46: 760-775.

14. Cumberlidge N (2008) Insular species of Afrotropical freshwater crabs (Crustacea : Decapoda : Brachyura : Potamonautidae and Potamidae) with special reference to Madagascar and the Seychelles. Contributions to Zoology 77: 71-81.

15. Daniels SR (2011) Reconstructing the colonisation and diversification history of the endemic freshwater crab (Seychellum alluaudi) in the granitic and volcanic Seychelles Archipelago. Molecular Phylogenetics and Evolution 61: 534-542.

16. Daniels SR, Cumberlidge N, Perez-Losada M, Marijnissen SAE, Crandall KA (2006) Evolution of Afrotropical freshwater crab lineages obscured by morphological convergence. Molecular Phylogenetics and Evolution 40: 227-235.

17. Dijkstra KDB (2007) Gone with the wind: westward dispersal across the Indian Ocean and island speciation in Hemicordulia dragonflies (Odonata : Corduliidae). Zootaxa: 27-48.

18. Fuchs J, Pons JM, Goodman SM, Bretagnolle V, Melo M, et al. (2008) Tracing the colonization history of the Indian Ocean scops-owls (Strigiformes : Otus) with further insight into the spatio-temporal origin of the Malagasy avifauna. Bmc Evolutionary Biology 8.

19. Gamble T, Bauer AM, Greenbaum E, Jackman TR (2008) Evidence for Gondwanan vicariance in an ancient clade of gecko lizards. Journal of Biogeography 35: 88-104.

20. Goodman SM, Buccas W, Naidoo T, Ratrimomanarivo F, Taylor PJ, et al. (2010) Patterns of morphological and genetic variation in western Indian Ocean members of the Chaerephon 'pumilus' complex (Chiroptera: Molossidae), with the description of a new species from Madagascar. Zootaxa: 1-36.

21. Goodman SM, Chan LM, Nowak MD, Yoder AD (2010) Phylogeny and biogeography of western Indian Ocean Rousettus (Chiroptera: Pteropodidae). Journal of Mammalogy 91: 593-606.

22. Goodman SM, Vuuren BJ, Ratrimomanarivo F, Probst JM, Bowie RCK (2008) Specific status of populations in the Mascarene Islands referred to *Mormopterus acetabulosus* (Chiroptera: Molossidae), with description of a new species. Journal of Mammalogy 89: 1316-1327.

23. Greenbaum E, Bauer AM, Jackman TR (2007) Homopholis and Blaesodactylus (Squamata : Gekkonidae) revisited: new insights from a molecular phylogeny. African Journal of Herpetology 56: 101-114.

24. Harmon LJ, Melville J, Larson A, Losos JB (2008) The Role of Geography and Ecological Opportunity in the Diversification of Day Geckos (Phelsuma). Systematic Biology 57: 562-573.

25. Heads M (2010) Evolution and biogeography of primates: a new model based on molecular phylogenetics, vicariance and plate tectonics. Zoologica Scripta 39: 107-127.

26. Herrmann M, Kienle S, Rochat J, Mayer WE, Sommer RJ (2010) Haplotype diversity of the nematode Pristionchus pacificus on Reunion in the Indian Ocean suggests multiple independent invasions. Biological Journal of the Linnean Society 100: 170-179.

27. Hume JP (2007) Reappraisal of the parrots (Aves : Psittacidae) from the Mascarene Islands, with comments on their ecology, morphology, and affinities. Zootaxa: 1-76.

28. Hundsdoerfer AK, Rubinoff D, Attie M, Wink M, Kitching IJ (2009) A revised molecular phylogeny of the globally distributed hawkmoth genus Hyles (Lepidoptera: Sphingidae), based on mitochondrial and nuclear DNA sequences. Molecular Phylogenetics and Evolution 52: 852-865.

29. Inoue JG, Kumazawa Y, Miya M, Nishida M (2009) The historical biogeography of the freshwater knifefishes using mitogenomic approaches: A Mesozoic origin of the Asian notopterids (Actinopterygii: Osteoglossomorpha). Molecular Phylogenetics and Evolution 51: 486-499.

30. Janssen T, Bystriakova N, Rakotondrainibe F, Coomes D, Labat JN, et al. (2008) Neoendemism in Madagascan scaly tree ferns results from recent, coincident diversification bursts. Evolution 62: 1876-1889.

31. Jonsson KA, Fjeldsa J (2006) Determining biogeographical patterns of dispersal and diversification in oscine passerine birds in Australia, Southeast Asia and Africa. Journal of Biogeography 33: 1155-1165.

32. Klaus S, Schubart CD, Brandis D (2006) Phylogeny, biogeography and a new taxonomy for the Gecarcinucoidea Rathbun, 1904 (Decapoda : Brachyura). Organisms Diversity & Evolution 6: 199-217.

33. Kohler F, Glaubrecht M (2010) Uncovering an overlooked radiation: molecular phylogeny and biogeography of Madagascar's endemic river snails (Caenogastropoda: Pachychilidae: Madagasikara gen. nov.). Biological Journal of the Linnean Society 99: 867-894.

34. Koopman MM, Baum DA (2008) Phylogeny and biogeography of tribe Hibisceae (Malvaceae) on Madagascar. Systematic Botany 33: 364-374.

35. Kreier HP, Schneider H (2006) Phylogeny and biogeography of the staghorn fern genus Platycerium (Polypodiaceae, Polypodiidae). American Journal of Botany 93: 217-225.

36. Kulju KKM, Sierra SEC, Draisma SGA, Samuel R, van Welzen PC (2007) Molecular phylogeny of Macaranga, Mallotus, and related genera (Euphorbiaceae s.s.): insights from plastid and nuclear DNA sequence data. American Journal of Botany 94: 1726-1743.

37. Kuntner M (2006) Phylogenetic systematics of the Gondwanan nephilid spider lineage Clitaetrinae (Araneae, Nephilidae). Zoologica Scripta 35: 19-62.

38. Kuntner M (2007) A monograph of Nephilengys, the pantropical 'hermit spiders' (Araneae, Nephilidae, Nephilinae). Systematic Entomology 32: 95-135.

39. Kuntner M, Agnarsson I (2011) Biogeography and diversification of hermit spiders on Indian Ocean islands (Nephilidae: Nephilengys). Molecular Phylogenetics and Evolution 59: 477-488.

40. Kuntner M, Agnarsson I (2011) Phylogeography of a successful aerial disperser: the golden orb spider Nephila on Indian Ocean islands. Bmc Evolutionary Biology 11.

41. Le Pechon T, Cao N, Dubuisson JY, Gigord LDB (2009) Systematics of Dombeyoideae (Malvaceae) in the Mascarene archipelago (Indian Ocean) inferred from morphology. Taxon 58: 519-531.

42. Le Pechon T, Dubuisson JY, Haevermans T, Cruaud C, Couloux A, et al. (2010) Multiple colonizations from Madagascar and converged acquisition of dioecy in the Mascarene Dombeyoideae (Malvaceae) as inferred from chloroplast and nuclear DNA sequence analyses. Annals of Botany 106: 343-357.

43. Legrand D, Chenel T, Campagne C, Lachaise D, Cariou ML (2011) Inter-island divergence within Drosophila mauritiana, a species of the D. simulans complex: Past history and/or speciation in progress? Molecular Ecology 20: 2787-2804.

44. Macey JR, Kuehl JV, Larson A, Robinson MD, Ugurtas IH, et al. (2008) Socotra Island the forgotten fragment of Gondwana: Unmasking chameleon lizard history with complete mitochondrial genomic data. Molecular Phylogenetics and Evolution 49: 1015-1018.

45. Manns U, Anderberg AA (2011) Biogeography of 'tropical Anagallis' (Myrsinaceae) inferred from nuclear and plastid DNA sequence data. Journal of Biogeography 38: 950-961.

46. Maurin O, Davis AP, Chester M, Mvungi EF, Jaufeerally-Fakim Y, et al. (2007) Towards a phylogeny for Coffea (Rubiaceae): Identifying well-supported lineages based on nuclear and plastid DNA sequences. Annals of Botany 100: 1565-1583.

47. Michalak I, Zhang LB, Renner SS (2010) Trans-Atlantic, trans-Pacific and trans-Indian Ocean dispersal in the small Gondwanan Laurales family Hernandiaceae. Journal of Biogeography 37: 1214-1226.

48. Micheneau C, Carlsward BS, Fay MF, Bytebier B, Pailler T, et al. (2008) Phylogenetics and biogeography of Mascarene angraecoid orchids (Vandeae, Orchidaceae). Molecular Phylogenetics and Evolution 46: 908-922.

49. Munchenberg T, Wollenberg KC, Glaw F, Vences M (2008) Molecular phylogeny and geographic variation of Malagasy iguanas (Oplurus and Chalarodon). Amphibia-Reptilia 29: 319-327.

50. Nagy ZT, Glaw F, Vences M (2010) Systematics of the snake genera Stenophis and Lycodryas from Madagascar and the Comoros. Zoologica Scripta 39: 426-435.

51. Nazari V, Larsen TB, Lees DC, Brattstrom O, Bouyer T, et al. (2011) Phylogenetic systematics of Colotis and associated genera (Lepidoptera: Pieridae): evolutionary and taxonomic implications. Journal of Zoological Systematics and Evolutionary Research 49: 204-215.

52. Noonan BP, Chippindale PT (2006) Vicariant origin of Malagasy reptiles supports late cretaceous antarctic land bridge. American Naturalist 168: 730-741.

53. O'Brien J, Mariani C, Olson L, Russell AL, Say L, et al. (2009) Multiple colonisations of the western Indian Ocean by Pteropus fruit bats (Megachiroptera: Pteropodidae): The furthest islands were colonised first. Molecular Phylogenetics and Evolution 51: 294-303.

54. Okajima Y, Kumazawa Y (2010) Mitochondrial genomes of acrodont lizards: timing of gene rearrangements and phylogenetic and biogeographic implications. Bmc Evolutionary Biology 10.

55. Pasquet E, Pons JM, Fuchs J, Cruaud C, Bretagnolle V (2007) Evolutionary history and biogeography of the drongos (Dicruridae), a tropical Old World clade of corvoid passerines. Molecular Phylogenetics and Evolution 45: 158-167.

56. Pokorny L, Olivan G, Shaw AJ (2011) Phylogeographic Patterns in Two Southern Hemisphere Species of Calyptrochaeta (Daltoniaceae, Bryophyta). Systematic Botany 36: 542-553.

57. Raselimanana AP, Noonan B, Karanth KP, Gauthier J, Yoder AD (2009) Phylogeny and evolution of Malagasy plated lizards. Molecular Phylogenetics and Evolution 50: 336-344.

58. Raxworthy CJ, Ingram CM, Rabibisoa N, Pearson RG (2007) Applications of ecological niche modeling for species delimitation: A review and empirical evaluation using day geckos (Phelsuma) from Madagascar. Systematic Biology 56: 907-923.

59. Rehan SM, Chapman TW, Craigie AI, Richards MH, Cooper SJB, et al. (2010) Molecular phylogeny of the small carpenter bees (Hymenoptera: Apidae: Ceratinini) indicates early and rapid global dispersal. Molecular Phylogenetics and Evolution 55: 1042-1054.

60. Renner SS, Strijk JS, Strasberg D, Thebaud C (2010) Biogeography of the Monimiaceae (Laurales): a role for East Gondwana and long-distance dispersal, but not West Gondwana. Journal of Biogeography 37: 1227-1238.

61. Rocha S, Carretero MA, Harris DJ (2010) Genetic diversity and phylogenetic relationships of Mabuya spp. (Squamata: Scincidae) from western Indian Ocean islands. Amphibia-Reptilia 31: 375-385.

62. Rocha S, Carretero MA, Vences M, Glaw F, Harris DJ (2006) Deciphering patterns of transoceanic dispersal: the evolutionary origin and biogeography of coastal lizards (Cryptoblepharus) in the Western Indian Ocean region. Journal of Biogeography 33: 13-22.

63. Rouhan G, Hanks JG, McClelland D, Moran RC (2007) Preliminary phylogenetic analysis of the fern genus Lomariopsis (Lomariopsidaceae). Brittonia 59: 115-128.

64. Rowson B, Tattersfield P, Symondson WOC (2011) Phylogeny and biogeography of tropical carnivorous land-snails (Pulmonata: Streptaxoidea) with particular reference to East Africa and the Indian Ocean. Zoologica Scripta 40: 85-98.

65. Russell AL, Goodman SM, Cox MP (2008) Coalescent analyses support multiple mainland-to-island dispersals in the evolution of Malagasy Triaenops bats (Chiroptera : Hipposideridae). Journal of Biogeography 35: 995-1003.

66. Schaefer H, Heibl C, Renner SS (2009) Gourds afloat: a dated phylogeny reveals an Asian origin of the gourd family (Cucurbitaceae) and numerous oversea dispersal events. Proceedings of the Royal Society B-Biological Sciences 276: 843-851.

67. Schwarz MP, Fuller S, Tierney SM, Cooper SJB (2006) Molecular phylogenetics of the exoneurine allodapine bees reveal an ancient and puzzling dispersal from Africa to Australia. Systematic Biology 55: 31-45.

68. Schweizer M, Seehausen O, Guntert M, Hertwig ST (2010) The evolutionary diversification of parrots supports a taxon pulse model with multiple trans-oceanic dispersal events and local radiations. Molecular Phylogenetics and Evolution 54: 984-994.

69. Sheldon FH, Lohman DJ, Lim HC, Zou F, Goodman SM, et al. (2009) Phylogeography of the magpie-robin species complex (Aves: Turdidae: Copsychus) reveals a Philippine species, an interesting isolating barrier and unusual dispersal patterns in the Indian Ocean and Southeast Asia. Journal of Biogeography 36: 1070-1083.

70. Sole CL, Scholtz CH (2010) Did dung beetles arise in Africa? A phylogenetic hypothesis based on five gene regions. Molecular Phylogenetics and Evolution 56: 631-641.

71. Sole CL, Wirta H, Forgie SA, Scholtz CH (2011) Origin of Madagascan Scarabaeini dung beetles (Coleoptera: Scarabaeidae): dispersal from Africa. Insect Systematics & Evolution 42: 29-40.

72. Stone RD, Andreasen K (2010) The Afro-Madagascan genus Warneckea (Melastomataceae): Molecular systematics and revised infrageneric classification. Taxon 59: 83-92.

73. Thiv M, Thulin M, Kilian N, Linder HP (2006) Eritreo-Arabian affinities of the Socotran flora as revealed from the molecular phylogeny of Aerva (Amaranthaceae). Systematic Botany 31: 560-570.

74. Thiv M, van der Niet T, Rutschmann F, Thulin M, Brune T, et al. (2011) Old-New world and trans-African dijunctions of Thamnosma (Rutaceae): intercontinental long-distance dispersal and local differentation in the succulent biome. American Journal of Botany 98: 76-87.

75. Tollenaere C, Brouat C, Duplantier JM, Rahalison L, Rahelinirina S, et al. (2010) Phylogeography of the introduced species Rattus rattus in the western Indian Ocean, with special emphasis on the colonization history of Madagascar. Journal of Biogeography 37: 398-410.

76. Toon A, Perez-Losada M, Schweitzer CE, Feldmann RM, Carlson M, et al. (2010) Gondwanan radiation of the Southern Hemisphere crayfishes (Decapoda: Parastacidae): evidence from fossils and molecules. Journal of Biogeography 37: 2275-2290.

77. Townsend TM, Tolley KA, Glaw F, Bohme W, Vences M (2011) Eastward from Africa: palaeocurrent-mediated chameleon dispersal to the Seychelles islands. Biology Letters 7: 225-228.

78. Trujillo RG, Patton JC, Schlitter DA, Bickham JW (2009) Molecular phylogenetics of the bat genus *Scotophilus* (Chiroptera: Vespertilionidae): perspectives from paternally and maternally inherited genomes. Journal of Mammalogy 90: 548-560.

79. Tsy J, Lumaret R, Mayne D, Vall AOM, Abutaba YIM, et al. (2009) Chloroplast DNA phylogeography suggests a West African centre of origin for the baobab, Adansonia digitata L. (Bombacoideae, Malvaceae). Molecular Ecology 18: 1707-1715.

80. Venkatasamy S, Khittoo G, Nowbuth P, Vencatasamy DR (2006) Phylogenetic relationships based on morphology among the Diospyros (Ebenaceae) species endemic to the Mascarene Islands. Botanical Journal of the Linnean Society 150: 307-313.

81. Verneau O, Du Preez LH, Laurent V, Raharivololoniaina L, Glaw F, et al. (2009) The double odyssey of Madagascan polystome flatworms leads to new insights on the origins of their amphibian hosts. Proceedings of the Royal Society B-Biological Sciences 276: 1575-1583.

82. Vidal N, Marin J, Morini M, Donnellan S, Branch WR, et al. (2010) Blindsnake evolutionary tree reveals long history on Gondwana. Biology Letters 6: 558-561.

83. Virgilio M, Delatte H, Backeljau T, De Meyer M (2010) Macrogeographic population structuring in the cosmopolitan agricultural pest Bactrocera cucurbitae (Diptera: Tephritidae). Molecular Ecology 19: 2713-2724.

84. Warren BH, Bermingham E, Prys-Jones RP, Thebaud C (2006) Immigration, species radiation and extinction in a highly diverse songbird lineage: white-eyes on Indian Ocean islands. Molecular Ecology 15: 3769-3786.

85. Wesener T, Raupach MJ, Sierwald P (2010) The origins of the giant pill-millipedes from Madagascar (Diplopoda: Sphaerotheriida: Arthrosphaeridae). Molecular Phylogenetics and Evolution 57: 1184-1193.

86. Wesener T, VandenSpiegel D (2009) A first phylogenetic analysis of Giant Pill-Millipedes (Diplopoda: Sphaerotheriida), a new model Gondwanan taxon, with special emphasis on island gigantism. Cladistics 25: 545-573.

87. Weyeneth N, Goodman SM, Appleton B, Wood R, Ruedi M (2011) Wings or winds: inferring bat migration in a stepping-stone archipelago. Journal of Evolutionary Biology 24: 1298-1306.

88. Weyeneth N, Goodman SM, Stanley WT, Ruedi M (2008) The biogeography of Miniopterus bats (Chiroptera: Miniopteridae) from the Comoro Archipelago inferred from mitochondrial DNA. Molecular Ecology 17: 5205-5219.

89. Wikstrom N, Avino M, Razafimandimbison SG, Bremer B (2010) Historical biogeography of the coffee family (Rubiaceae, Gentianales) in Madagascar: case studies from the tribes Knoxieae, Naucleeae, Paederieae and Vanguerieae. Journal of Biogeography 37: 1094-1113.

90. Wright TF, Schirtzinger EE, Matsumoto T, Eberhard JR, Graves GR, et al. (2008) A multilocus molecular phylogeny of the parrots (Psittaciformes): Support for a Gondwanan origin during the Cretaceous. Molecular Biology and Evolution 25: 2141-2156.

91. Yassin A, Araripe LO, Capy P, Da Lage JL, Klaczko LB, et al. (2008) Grafting the molecular phylogenetic tree with morphological branches to reconstruct the evolutionary history of the genus Zaprionus (Diptera : Drosophilidae). Molecular Phylogenetics and Evolution 47: 903-915.

92. Zhang P, Wake MH (2009) A mitogenomic perspective on the phylogeny and biogeography of living caecilians (Amphibia: Gymnophiona). Molecular Phylogenetics and Evolution 53: 479-491.

93. Zimmermann NFA, Ritz CM, Hellwig FH (2010) Further support for the phylogenetic relationships within Euphorbia L. (Euphorbiaceae) from nrITS and trnL-trnF IGS sequence data. Plant Systematics and Evolution 286: 39-58.

94. Zuccon D, Pasquet E, Ericson PGP (2008) Phylogenetic relationships among Palearctic-Oriental starlings and mynas (genera Sturnus and Acridotheres : Sturnidae). Zoologica Scripta 37: 469-481.
