# Supplementary material for: A Multi-Clade Test Supports the Intermediate Dispersal Model of Biogeography
Source: PLoS One. 2014 Jan 21;9(1):e86780. doi: 10.1371/journal.pone.0086780 (PMC3897756; doi:10.1371/journal.pone.0086780)
Supplement: Appendix S2 — Results of regression analyses in R. (DOCX) [file pone.0086780.s002.docx]

Linear result.

Call:

lm(formula = W ~ DE, data = D)

Residuals:

Min 1Q Median 3Q Max

-133.754 -25.679 2.936 20.744 259.518

Coefficients:

Estimate Std. Error t value Pr(>|t|)

(Intercept) -20.744 6.695 -3.099 0.00256 **

DE 18.808 1.286 14.625 < 2e-16 ***

---

Signif. codes: 0 ‘***’ 0.001 ‘**’ 0.01 ‘*’ 0.05 ‘.’ 0.1 ‘ ’ 1

Residual standard error: 54.43 on 95 degrees of freedom

Multiple R-squared: 0.6925, Adjusted R-squared: 0.6892

F-statistic: 213.9 on 1 and 95 DF, p-value: < 2.2e-16

Polynomial result

> summary(lmP)

Call:

lm(formula = W ~ DE + I(DE^2), data = D)

Residuals:

Min 1Q Median 3Q Max

-63.275 -11.858 -10.472 -3.005 251.995

Coefficients:

Estimate Std. Error t value Pr(>|t|)

(Intercept) 12.85836 5.72411 2.246 0.027 *

DE -1.01025 2.15560 -0.469 0.640

I(DE^2) 0.90858 0.08991 10.106 <2e-16 ***

---

Signif. codes: 0 ‘***’ 0.001 ‘**’ 0.01 ‘*’ 0.05 ‘.’ 0.1 ‘ ’ 1

Residual standard error: 37.88 on 94 degrees of freedom

Multiple R-squared: 0.8526, Adjusted R-squared: 0.8495

F-statistic: 271.9 on 2 and 94 DF, p-value: < 2.2e-16

Model comparison

> anova(lml,lmP)

Analysis of Variance Table

Model 1: W ~ DE

Model 2: W ~ DE + I(DE^2)

Res.Df RSS Df Sum of Sq F Pr(>F)

1 95 281462

2 94 134899 1 146563 102.13 < 2.2e-16 ***

---

Signif. codes: 0 ‘***’ 0.001 ‘**’ 0.01 ‘*’ 0.05 ‘.’ 0.1 ‘ ’ 1

>
